# Rapid HIV Viral Load Suppression in those Initiating Antiretroviral Therapy at First Visit after HIV Diagnosis

**DOI:** 10.1038/srep32947

**Published:** 2016-09-06

**Authors:** Martin Hoenigl, Antoine Chaillon, David J. Moore, Sheldon R. Morris, Sanjay R. Mehta, Sara Gianella, K. Rivet Amico, Susan J. Little

**Affiliations:** 1 Division of Infectious Diseases, Department of Medicine, University of California San Diego, San Diego, California, United States; 2 Section of Infectious Diseases and Tropical Medicine, Department of Internal Medicine, Medical University of Graz, Graz, Austria; 3 Division of Pulmonology, Department of Internal Medicine, Medical University of Graz, Graz, Austria; 4 Department of Psychiatry, University of California San Diego (UCSD), La Jolla, California, United States; 5 Veterans Affairs Healthcare System, San Diego, California, United States; 6 Department of Health Behavior and Health Education, School of Public Health, University of Michigan, Ann Arbor, MI, USA

## Abstract

Expert guidelines for antiretroviral therapy (ART) now recommend ART as soon as possible in all HIV infected persons to reduce the risk of disease progression and prevent transmission. The goal of this observational study was to evaluate the impact of very early ART initiation and regimen type on time to viral suppression. We evaluated time to viral suppression among 86 persons with newly-diagnosed HIV infection who initiated ART within 30 days of diagnosis. A total of 36 (42%) had acute, 27 (31%) early, and 23 (27%) had established HIV infection. The median time from an offer of immediate ART to starting ART was 8 days. A total of 56/86 (65%) initiated an integrase inhibitor-based regimen and 30/86 (35%) a protease inhibitor-based regimen. The time to viral suppression was significantly shorter in those receiving an integrase inhibitor- versus a protease inhibitor-based regimen (p = 0.022). Twenty-two (26%) initiated ART at their HIV care intake visit and 79% of these participants achieved viral suppression at week 12, 82% at week 24 and 88% at week 48. ART initiated at the intake visit led to rapid and reliable viral suppression in acute, early and chronic HIV infection, in particular when integrase inhibitor-based regimens were used.

Despite intense efforts to diagnose HIV infection as early as possible, engage newly-diagnosed individuals into care, and recommend antiretroviral therapy (ART) for all infected persons^1^,^2^, HIV incidence still remains stable in the United States, and is increasing among men who have sex with men (MSM)^3^,^4^. Universal treatment as prevention (TasP) is one of the most promising strategies to reduce HIV incidence^5^,^6^,^7^. TasP may be particularly effective when initiated during acute HIV infection (AHI), which is associated with transient levels of extremely high titer viremia^8^,^9^. AHI therefore serves as a major driver of HIV transmission in sexually active populations, and in particular among MSM in the United States and other resource rich countries^10^,^11^,^12^. As many as half of HIV transmissions occur from persons with AHI^13^. Very early initiation of ART in AHI may rapidly decrease viral loads and therefore reduce infectiousness during this particularly important period. There is also consistent evidence that very early ART may benefit the individual infected with HIV by leading to more rapid and robust immunologic recovery, lower inflammation and reduced viral reservoir size compared to a later start^14^,^15^,^16^,^17^,^18^.

ART as early as possible after diagnosis improves morbidity and mortality in all stages of HIV infection^8^,^19^. Expert guidelines for ART therefore now recommend ART as soon as possible, regardless of CD4 cell count, to reduce the risk of disease progression and prevent HIV transmission^1^. Limited data exist, however, on the uptake and barriers to the initiation of very early ART, in particular about ART delivered as early as the day an individual is informed about their HIV diagnosis. Importantly, newly HIV diagnosed persons are faced with negotiating a complex healthcare system while coping with acute negative reactions (e.g., fear, anxiety, depression, stigma and isolation) that can erode investment in engaging in ART and may theoretically result in an unfavourable outcomes of early ART^20^,^21^,^22^.

The goal of this study was to evaluate the impact of early ART and regimen type on time to viral suppression, with a particular focus on ART initiated on the same-day of HIV care initiation.

## Results

### Demographic characteristics

A total of 86 persons with newly-diagnosed HIV infection and early ART initiation (i.e., within 30 days of diagnosis) were included in this analysis. Overall, 84 (98%) were male and two (2%) were transgender females. A total of 82/84 (98%) men and both transgender females reported sex with men, 2/84 men (both MSM) also reported injection drug use and 8/84 men (including 6/82 MSM) sex with women. Median age was 32 years (range 20–66 years). Race/Ethnicity was reported by 82/86 (95%) of study participants: 30 (37%) Hispanic ethnicity, 39 (48%) White race, 8 (10%) Black race and 5 (6%) Asian race.

### ART initiation, Viral Loads, CD4 and CD8 counts

Thirty-six of 86 participants (42%) were diagnosed with AHI, 27 (31%) early HIV infection, and 23 (27%) with established HIV infection (i.e., infection duration >170 days). The median time form estimated date of infection (EDI) to ART start in those with acute or early HIV infection was 32 days (range 10–194 days). Median time from EDI to ART start for those with AHI was 25 days (range 10–40 days), and 91 days (range 36 to 194 days) for those with early infection (Table 1). Choice of immediate ART regimen was unrestricted and preferred regimen was selected based on participant/provider preference. All individuals received a regimen recommended by treatment guidelines that included a dual nucleoside reverse-transcriptase inhibitor (NRTI; either tenofovir alafenamide/emtricitabine or tenofovir disoproxil/emtricitabine), combined with either an integrase strand transfer inhibitor (INSTI, cobicistat boosted elitegravir plus NNRTI as a fixed dose combination [FDC] pill; 56/86, 65%), or a protease inhibitor (ritonavir boosted atazanavir in 23/86, ritonavir boosted darunavir in 7/86; total 30/86, 35%).

Overall, median time from HIV diagnosis to ART start was 13 days (range 0–29 days), and median time from the first clinic intake to start of ART was 8 days (IQR 0–28 days). Table 2 shows median days from diagnosis and first visit to ART start for those with AHI (samples had to be sent out for NAT testing and participants then reached for arranging follow-up visits), those with early infection (point-of-care HIV diagnosis), and those with chronic HIV infection (point-of-care HIV diagnosis), and also those with INSTI-based ART regimen and those with protease inhibitor-based regimen.

A total of 79 participants (92%) completed a week 12 follow-up visit and 49/79 (62%) were virally suppressed at that time. At week 24, 59/70 (84%) were virally suppressed, while at week 48 55/60 (92%) were virally suppressed (as the majority of participants initiated ART in 2015, 81% of study participants completed the 24 week visit and 70% the 48 week visit at the time the analysis was performed). Viral suppression rates at week 24 and 48 did not differ significantly between those diagnosed with AHI, early or chronic HIV infection (Table 2). Median time to documented viral suppression was 12 weeks (IQR 4–24 weeks) and did not differ between those with AHI, early or chronic HIV (AHI median 12 weeks, IQR 12–24 weeks; early HIV median 12 weeks, IQR 4–24 weeks; chronic HIV median 12 weeks, IQR 4–12 weeks; n.s., Kruskal Wallis Test). A significantly shorter time to viral suppression was observed in those receiving INSTI-based regimen, compared to those with protease inhibitor-based regimens (median weeks to viral suppression 12, IQR 4–24 weeks in those with INSTI vs. median 24, IQR 12–24 in those with protease inhibitors; p = 0.022, Mann-Whitney U Test; baseline viral loads did not differ between those two groups).

Overall, CD4/CD8 cell ratios increased significantly between baseline and the final follow-up between weeks 24 and 48 (n = 75; median 0.57, IQR 0.31–0.99 at baseline vs. median 1.01, IQR 0.69–1.24 at final visits; p < 0.001, Wilcoxon signed rank test; Table 2). While CD4 cells increased significantly, from median 466 cells/mL (IQR 329–614) at baseline to median 721 cells/mL (IQR 512–962) at the final visit (p < 0.001), there were no significant differences in CD8 cell counts (p = 0.346).

### Within a week vs. later ART start

A total of 41/86 (48%) of participants initiated ART within one week of their first clinic intake (15 with acute, 13 with early and 13 with chronic infection), while 45 (52%) initiated ART between one and four weeks after their first clinic intake (21 with AHI, 14 with early and 10 with chronic infection). Age (p = 0.484, Mann Whitney U test) and race/ethnicity (p = 0.491, Chi Squared test) did not differ between those who initiated ART within a week of their first visit and those who initiated ART between one to four weeks after their first visit. Baseline viral loads did not differ between those who started early versus later. At week 12 of ART, those who started ART within a week of being offered ART had a significantly greater reduction of viral loads compared to those who started after one week of being offered ART (p = 0.006, Mann Whitney U test) and there was a trend towards more frequently observed viral suppression (27/37, 73% vs. 22/42, 50%; p = 0.060 Chi Squared test) when compared to those who started later. However, those who started ART within a week received more often INSTI based regimens, and when only individuals receiving INSTI were analyzed there was no difference in reduction of viral loads or viral suppression at week 12 between those who started within a week versus those who started later. No difference in the rate of virologic suppression was observed at week 24 and 48. Overall, time to viral suppression did not differ between those who started within a week and those who started later.

### Same-day ART initiation

Twenty-two (26% of the whole study population) initiated ART during their first visit; ten (45%) with acute, four (18%) with early and eight (36%) with chronic HIV infection. Age (p = 0.601), race (p = 0.738), gender (p = 0.065) as well as baseline viral loads (p = 0.607) and CD4/CD8 ratios (p = 0.933) did not differ from those who started ART later. Those who initiated ART on day 0 had a trend towards being more likely virally suppressed at week 12 of ART (79% virally suppressed of those with same-day ART vs. 57% of the remaining study population; p = 0.068; Fishers exact test). No difference was observed at week 24 (18/22, 82% virally suppressed) and week 48 (15/17, 88% virally suppressed), and also time to viral suppression did not differ between those with same-day ART and those who initiated ART later.

## Discussion

We evaluated the impact of early ART on time to viral suppression, and found that ART initiated within 30 days of diagnosis led to rapid and reliable viral suppression in acute, early and chronic HIV infection, in particular when INSTI-based regimens were used. A quarter of the study population initiated ART at their first clinic intake, and 79% of those individuals achieved viral suppression as early as week 12.

Importantly, 42% of individuals analyzed here and 45% of those with same-day ART had acute HIV infection. Uptake of same-day ART (e.g., of those with AHI 28% initiated same-day ART and 42% ART within a week), was lower in this study than preliminary results reported by the RAPID study^23^. Once ART was initiated, time to viral suppression was comparable between our study and the RAPID study. Both studies indicate that very early ART may rapidly reduce viral loads and thus significantly reduce infectiousness.

Identifying strategies to reduce the incidence of HIV, especially in MSM populations, remains one of the highest NIH public health priorities; 63% of HIV transmissions are attributed to MSM and 78% of new infections in the US are among males^24^. While early ART may be one of the most promising strategies to reduce HIV incidence, the extent to which rapid suppression of virus impact subsequent HIV transmission in the early phases after HIV diagnosis remains unknown, as diagnosis of HIV infection is often associated with at least transiently reduced risk behaviors and a decreased risk of HIV transmission even in the absence of ART^11^. In addition to reducing transmission risk, same-day ART in AHI may also limit the size of viral reservoirs and hyper-infectivity^25^.

Limitations of our study include the single-center retrospective design and relatively small sample size, particularly in subgroups. Furthermore, our study cohort consisted nearly exclusively of MSM and may not be generalizable to other HIV-infected subpopulations beyond MSM. With that said, our study cohort is representative of the San Diego HIV epidemic, where MSM bear by far the greatest HIV burden^26^.

In conclusion, our findings indicate that early ART is associated with rapid viral suppression. Given that little is presently known about how individuals react to immediate ART, we are presently ill prepared to provide the social, structural or behavioral support that could optimize patient success. Future studies are needed to elucidate factors influencing acceptance of same-day access to ART and to develop infrastructure support for early initiation and continued maintenance of ART.

## Methods

In this observational study, we analyzed 86 persons with newly-diagnosed HIV infection between August 2010 and December 2015 who were enrolled in the San Diego Primary Infection Resource Consortium (SD PIRC) and initiated ART within 30 days of diagnosis.

Study participants were screened using the “Early Test”, a community-based, confidential HIV testing program^27^,^28^ that provides free, point of care rapid HIV serologic testing followed by routine reflex to individual donation HIV nucleic acid amplification testing (NAT) in all antibody (Ab)-negative persons. Positive HIV NAT results are provided at a return visit as soon as possible after test results are available^26^,^27^,^29^. EDIs were calculated for all (n = 63) recently-infected persons using previously published serologic and virologic criteria^14^. AHI was defined by a negative or indeterminate HIV Ab test in the presence of detectable HIV-1 RNA, corresponding to Fiebig stages I-II, with mean EDI within the last 10 days (95%CI interval 7–14 days)^30^. Early HIV infection was defined as HIV Ab+/detuned HIV Ab consistent with infection <170 days^31^,^32^,^33^,^34^.

Individuals with positive HIV rapid test results, confirmed with a second (different) HIV rapid test assay (early and chronic HIV infections) were informed of their HIV diagnosis at the time of testing. Individuals diagnosed with AHI were informed about their HIV diagnosis within a median of 4 days (interquartile range [IQR] 3–6 days) after HIV NAT testing. Clinical laboratories including CD4 cell count and HIV RNA were performed at the first HIV care intake visit after HIV diagnosis. Immediate, free of charge, ART was offered at the first intake visit. Routine follow-up visits were performed in study participants with laboratory assessments at weeks 4, 12, 24 and 48 in all persons who initiated ART. As 44/86 participants (51%) initiated ART in 2015, 48-week follow-up data were only available in a subset of participants. HIV RNA < 50 copies/ml was defined as virally suppressed.

While free of charge immediate ART was offered sporadically between 2010 and June 2014, it was offered to all newly-diagnosed individuals since June 2014. Therefore, two thirds of participants (57; 66%) initiated ART between June 2014 and December 2015 (immediate ART was offered to 77 individuals during this time period, and 74% of those initiated ART within 30 days of diagnosis).

For statistical analysis, SPSS version 23 (SPSS, Inc., Chicago, IL, USA) was used. The impact of regimen type, stage of HIV infection at diagnosis, and timing of ART initiation (i.e. within 7 days and same-day of first clinic intake versus later) on viral suppression suppression was evaluated. Wilcoxon signed rank test was used for analysis of repeated measures and Fishers Exact/Chi-Squared test or Mann-Whitney U test or Kruskal Wallace test for comparison of demographics, baseline viral loads and CD4 counts, reductions of viral loads and time to viral suppression. The University of California San Diego’s Human Research Protections Program approved the study protocol, consent process, and all study-related procedures. All study participants provided voluntary, written informed consent before any study procedures were undertaken. The methods were carried out in accordance with the approved guidelines.

## Additional Information

**How to cite this article**: Hoenigl, M. *et al*. Rapid HIV Viral Load Suppression in those Initiating Antiretroviral Therapy at First Visit after HIV Diagnosis. *Sci. Rep.* **6**, 32947; doi: 10.1038/srep32947 (2016).

## Figures and Tables

**Table 1:** Time from estimated date of infection (EDI) to ART initiation and CD4/CD8 cell ratios, CD4, and CD8 counts at baseline and week 24–48 follow up visits after initiating ART.

Characteristics and Time points	Number of individuals	Time from EDI to ART initiation (days, range)	Baseline CD4/CD8 cell ratio (median, range)	Week 24-48 CD4/CD8 cell ratio (median, range)	p-value (Wilcoxons signed rank test)	Baseline CD4 cell count, cells/mm^3^ (median, range)	Week 24-48 CD4 cell count, cells/mm^3^ (median, range)	p-value (Wilcoxons signed rank test)	Baseline CD8 cell count, cells/mm^3^ (median, range)	Week 24–48 CD8 cell count, cells/mm^3^ (median, range)	p-value (Wilcoxons signed rank test)
Acute HIV infection	36 (42%)	25 (10–40)	0.59 (0.07–2.60)	1.15 (0.48–3.97; n = 29)	<0.001	456 (67–1113)	827 (283–1539; n = 29)	<0.001	744 (110–6055)	791 (338–1476; n = 29)	n.s.
Early HIV infection	27 (31%)	91 (36–194)	0.65 (0.06–1.45)	1.02 (0.42–1.79; n = 24)	0.001	590 (166–973)	743 (434–1219; n = 24)	0.002	830 (299–8777)	843 (299–1264; n = 24)	n.s.
Chronic HIV infection	23 (27%)	—	0.42 (0.17–1.26)	0.81 (0.28–2.04; n = 22)	<0.001	377 (111–958)	597 (255–1523; n = 22)	<0.001	681 (378–2363)	759 (298–1508; n = 22)	n.s.

**Table 2:** Time to antiretroviral Therapy (ART) and viral loads at baseline and follow up visits after ART initiation in individuals with newly diagnosed acute, early or chronic HIV infection, and those who initiated integrase inhibitor versus protease inhibitor based ART regimen.

Characteristics and Time points	Number of individuals (n, %)	Time from Diagnosis to ART initiation (days, range)	Time from HIV care intake visit to ART initiation (days, range)	Baseline Viral load log10 RNA (median, range)	Week 4 Viral load log10 RNA (median, range)	Week 4 Virally suppressed (n, %)	Week 12 Viral load log10 RNA (median, range)	Week 12 Virally suppressed (n, %)	Week 24 Viral load log10 RNA (median, range)	Week 24 Virally suppressed (n, %)	Week 48 Viral load log10 RNA (median, range)	Week 48 Virally suppressed
Acute HIV infection	36 (42%)	14 (0–29)	10 (0–23)	5.74 (1.89–7.48)	2.46 (1.30–5.01)	7 (19%)	1.75 (1.30–4.15; n = 33)	16 (48%)	1.32 (1.30–2.26; n = 25)	20 (80%)	1.30 (1.09–4.98; n = 25)	22 (88%)
Early HIV infection	27 (31%)	13 (0–28)	8 (0–28)	4.65 (2.89–7.00)	1.92 (1.30–.67; n = 25)	10 (40%)	1.36 (1.30–2.69; n = 26)	15 (58%)	1.30 (1.30–3.91; n = 23)	21 (91%)	1.43 (1.30–4.59; n = 21)	19 (90%)
Chronic HIV infection	23 (27%)	9 (0–21)	7 (0–14)	4.54 (2.48–6.47)	1.85 (1.30–3.41; n = 22)	9 (41%)	1.30 (1.30–1.96; n = 20)	18 (90%)	1.30 (1.30–2.04; n = 18)	18 (82%)	1.30 (1.30–1.60; n = 14)	14 (100%)
Integrase inhibitor-based ART Regimen	56 (65%)	9 (0–29)	6 (0–28)	4.80 (1.89–7.00)	1.90 (1.30–3.78; n = 53)	20 (38%)	1.30 (1.30–2.85; n = 50)	38 (76%)	1.30 (1.30–3.91; n = 49)	43 (88%)	1.30 (1.09–4.98; n = 36)	33 (92%)
Protease inhibitor-based ART Regimen	30 (35%)	16 (0–22)	13 (0–21)	5.22 (2.89–7.48)	2.80 (1.30–5.02)	6 (20%)	2.11 (1.30–4.15; n = 29)	11 (21%)	1.68 (1.30–2.26; n = 21)	16 (76%)	1.68 (1.30–1.91; n = 24)	22 (92%)
